# A Smartphone Game to Prevent HIV Among Young Africans: Protocol for a Randomized Pilot Study of a Mobile Intervention

**DOI:** 10.2196/11209

**Published:** 2019-03-27

**Authors:** Gaëlle Sabben, Victor Akelo, Victor Mudhune, Ken Ondeng'e, Richard Ndivo, Rob Stephenson, Kate Winskell

**Affiliations:** 1 Hubert Department of Global Health Rollins School of Public Health Emory University Atlanta, GA United States; 2 HIV Research Branch Centre for Global Health Research Kenya Medical Research Institute Kisumu Kenya; 3 The Center for Sexuality and Health Disparities School of Nursing University of Michigan Ann Arbor, MI United States

**Keywords:** HIV, youth, sub-Saharan Africa, Kenya, serious game, narrative, mobile phone, pilot test, randomized controlled trial, mhealth, prevention, smartphone

## Abstract

**Background:**

Young people aged under 25 years make up an increasing proportion of the population in emerging economies such as Kenya, where half of new adult HIV infections are among 15- to 24-year olds. Interventions targeting this age group have the potential to avert HIV infections among an increasingly large at-risk population. Interactive communication technologies offer a promising platform for reaching young people in engaging ways.

**Objective:**

*Tumaini* is a narrative-based smartphone game designed to help young Africans protect themselves from HIV. The objective of this study was to pilot test the game, focusing on the data needed to inform a future randomized controlled efficacy trial, including assessments of study feasibility and safety.

**Methods:**

The study took place in Kisumu Town, western Kenya, in spring 2017. The game-based intervention was pilot tested for 16 days with a sample of 60 preadolescents aged 11 to 14 years. Participant recruitment was initiated through schools. Participants were randomly assigned to the control or intervention arms of the study. One parent for each of the intervention arm participants was also recruited (n=30). The intervention arm participants were provided with smartphones on which *Tumaini* was loaded so that they could play the game at home. Youth completed behavioral surveys at baseline, posttest, and 6-week follow-up. The intervention arm participants provided quantitative feedback on their experience of the game-based intervention at posttest. They and their parents further participated in postintervention focus group discussions. Feasibility-related study metrics were collected on recruitment, enrollment, attrition, safety of participants, and return of phones.

**Results:**

Recruitment and enrollment of the 60 preadolescents and parents were successfully completed within 18 days. No participants were lost to follow-up: all youth completed all 3 waves of the survey and 27 intervention arm youth and 22 parents and caregivers participated in the focus groups. No safety concerns were reported. All phones were returned after the intervention period; none were damaged or lost. All intervention arm participants initiated gameplay, recording a mean exposure time just under 27 hours.

**Conclusions:**

Findings indicate that it is feasible and safe to test a smartphone-based HIV prevention intervention for very young adolescents in urban and peri-urban sub-Saharan Africa by initiating recruitment in schools and temporarily providing youth participants with smartphones on which the game is loaded. A randomized controlled trial powered to assess the efficacy of the game-based intervention is being designed to be carried out in the same geographic area as the pilot, using similar methods.

**Trial Registration:**

ClinicalTrials.gov NCT03054051; https://clinicaltrials.gov/ct2/show/NCT03054051 (Archived by WebCite at http://www.webcitation.org/6wjwpX8Bg.)

**International Registered Report Identifier (IRRID):**

DERR1-10.2196/11209

## Introduction

In 2016, 1.8 million people around the world became infected with HIV, adding to the more than 35 million already living with the virus. More than 2 in every 5 infections occur in southern and eastern Africa. Youth aged 15 to 24 years continue to account for a large proportion of the population becoming infected, and young women are especially at risk [1]. Due to demographic shifts, adolescents make up an increasingly large proportion of the population in emerging economies, with 60% of the population in sub-Saharan Africa currently under 25 years [2,3]. It is projected that globally, under current conditions, an additional 3.5 million adolescents will become HIV-positive by 2030 [4].

Kenya and neighboring Tanzania and Uganda account for 21% of all HIV infections among 15- to 19-year olds in sub-Saharan Africa [5]. In Kenya, youth infections continue to increase. In 2015, young people aged 15 to 24 years accounted for over half of all new adult infections, with young women outnumbering young men 2:1 [6].

There is growing recognition that programs specifically targeting young people are critical to reducing the burden of HIV among adolescents and the population in general [7,8]. To maximize the impact of these interventions on infection rates, it is important to reach young people before they engage in behaviors that put them at risk for HIV. In addition, a strong foundation of accurate knowledge, positive attitudes and perceived social norms, healthy behavioral intentions, parent-child communication, and behavioral skills and related self-efficacy to avoid or reduce risk can help young people protect their health once they reach sexual debut and beyond [9-14]. Mobile technology can be leveraged to address these needs [15].

Increasingly accessible mobile technologies offer a promising platform for delivering interactive health promotion interventions that reach youth in new and engaging ways. In Kenya, 90% of the population has access to a mobile phone [16], and smartphone use is on the rise, as handsets become more accessible and affordable [17]. Increasing engagement with smart technologies affords young people growing opportunities to learn and practice skills where they are, when they want, and as often as they need. These technologies have the potential to deliver important information and skills training in highly interactive ways at lower cost and greater reach than is possible with group-based prevention interventions; however, further research is needed to determine the extent to which they are able to deliver on this promise [18]. In addition, it is possible for the intervention to be delivered with near-perfect fidelity, allowing only for intended, programmed, and user-driven customization [19,20].

The growing interest in mobile health (mHealth) and the recognition of its potential has led to the development of a range of interventions tackling a variety of health issues, including sexual and reproductive health. In particular, there is increasing recognition that serious electronic games, where the primary purpose is not entertainment [21], can deliver content in intrinsically motivating ways, blending entertainment with key content and skills training. Even more importantly, by giving players the opportunity to inhabit and navigate new situations, such interventions offer an especially exciting avenue for cognitive rehearsal and experiential skills building.

Although there is a growing evidence base for the efficacy of mHealth among adolescents [22], many mHealth interventions, including those targeting behavior change around sexual and reproductive health and developed for use in low-income countries, have thus far been delivered via feature phone, or nonsmartphone, platforms, for example, 2-way messaging interventions [23,24]. A smartphone-based mode of delivery provides access to more interactive and engaging interventions, including serious games. Internet connectivity, either via Wi-Fi or a data plan, can allow users to find, download, and update intervention materials as needed. It also makes it possible for new content and messages to be pushed to users and for user activity and progress to be monitored remotely. In addition, the large storage capacity of smartphones provides space for a large complex program that allows for significant interaction. The traditionally larger screen also provides a more attractive learning environment. These features make smartphones a particularly appealing delivery platform for game-based interventions, without the user cost and accessibility challenges of a computer-based or tablet-based program.

Smartphones are a relatively new, though promising [25,26], platform for delivering theory-driven health promotion interventions. Thus, few such interventions have been efficacy tested, particularly those focusing on sexual and reproductive health, and aimed at adolescents in low-resource settings. If we are to ensure that those at risk have access to the best skills-building and informational content available, we must thoroughly test and evaluate promising new approaches by tailoring proven research methods to the needs of these new platforms and interventions.

This paper describes the protocol for the pilot study of a narrative-based smartphone game called *Tumaini* in Kisumu, western Kenya. This study was carried out in collaboration with the Kenya Medical Research Institute (KEMRI). Its objective was to pilot test the game-based intervention, focusing on data needed for a larger efficacy trial, including assessments of study feasibility and safety, where feasibility is defined as successful recruitment, enrollment, and retention of a cohort of 11- to 14-year olds and safety is defined as absence of negative outcomes from participation in the study. The purpose of this paper is to present our protocol, as well as the study’s feasibility and safety findings, with a view to guiding the development of other smartphone-based mHealth intervention studies aimed at school-aged populations in low-resource settings.

## Methods

### Overview

This study was a pilot randomized controlled trial (RCT) conducted with 60 male and female preadolescents aged 11 to 14 years, in the East, West, and Central administrative locations of Kisumu Town, Kisumu County, Kenya. Participants were identified via school-initiated recruitment and randomized to 1 of the 2 study arms. Adolescents in the control arm received standard of care (no intervention beyond any existing sex education from family, school, and peers); intervention arm participants played *Tumaini*, an interactive, narrative-based electronic game, which had been loaded on study-provided, low-cost Android smartphones. The intervention period lasted 16 days. Participants in both arms completed baseline, postintervention, and follow-up surveys. Intervention arm adolescents and their parents also participated in postintervention focus group discussions (FGDs). Primary outcomes for this study focused on the feasibility and safety of the study. Secondary outcomes included acceptability of the game and behavioral mediators of sexual initiation and condom use, reported elsewhere [27].

### Description of the Intervention

*Tumaini* (Multimedia Appendix 1) is a theoretically grounded, narrative-based game for inexpensive Android smartphones developed in collaboration with a US commercial game developer, Realtime Associates, and with input both from US-based and Kenyan specialists in adolescent sexual health and from Kenyan preadolescents and their parents. It is designed to increase age and condom use at first sex by increasing knowledge about sexual health and HIV, building risk-avoidance and risk-reduction skills and related self-efficacy, challenging HIV stigma and harmful gender norms and attitudes, fostering future orientation, goal-setting, and planning, and promoting dialogue with adult mentors. *Tumaini* uses interactive narrative to promote problem-solving, cognitive and behavioral rehearsal, observational learning, and immersion.

*Tumaini’*s design draws on social behavioral theory, including Social Cognitive Theory [28] and the Theory of Possible Selves [29], Entertainment-Education literature [30], games for health literature [31-34], and existing evidence-based HIV prevention interventions aimed at youth [35-39]. Game design and scripting also drew on extensive research on a vast sample of HIV-themed narratives written by young Africans [40-42]. Preadolescents and parents in Kisumu provided input via FGDs. The game is in English and includes an audio track featuring Kenyan voice talent.

The mobile intervention is made up of 3 intersecting components: (1) a choose-your-own-adventure game, where players role-play 6 diverse characters, making choices for them that determine the course of their lives, (2) a set of mini games that reinforce knowledge and skills development, and (3) *My Story,* in which players create an avatar of themselves, set personal goals, and relate the game narrative to their own lives. *Tumaini* comprises approximately 12 hours of discrete gameplay and is designed to be replayed so that players can observe the outcomes of different decisions.

### Trial Registration, Ethics, Consent, and Institutional Board Approval

The study was approved by the Institutional Review Boards of Emory University (IRB00081150) and KEMRI (KEMRI/SERU/CGHR/019/3100). The study was also registered on ClinicalTrials.gov (NCT03054051).

### Eligibility

Preadolescents eligible to enroll had to meet 4 criteria: (1) be aged 11 to 14 years at the time of recruitment, (2) reside in Kisumu Town, (3) not attend a boarding school, and (4) demonstrate a Grade 3 to 4 English proficiency on the Flesch-Kincaid scale, assessed via a brief screening exercise. This level of English literacy was not expected to act as a barrier to recruitment, as primary school education (ages 6 to 14 years) has been free and compulsory in Kenya since 2003, and all subjects are taught in English from Standard 4 (age 10 years and above) onward. Boarding school students were ineligible as they do not return home on weekends during the school term and would thus have been unavailable for the follow-up surveys and postintervention FGDs. Only 1 child per family was eligible to enroll. Any preadolescent or parent in a family previously engaged in formative research or review of the game or study documents was ineligible.

Parents were eligible to participate in postintervention focus groups if they had a child enrolled in the study.

Smartphone ownership was not a requirement for eligibility as phones were provided to intervention arm participants. This strategy sought to minimize socioeconomic bias in the sample. It also ensured consistency of smartphone technology.

### Incentives

Participants were not offered incentives for this study. They were reimbursed US $5 for their time and transportation costs for each study visit.

### Recruitment

The KEMRI study lead contacted county Ministry of Education officials for permission to initiate recruitment of participants through schools in the Kisumu East, Central, and West subcounties. In dialogue with the Emory team, the KEMRI team randomly sampled 11 schools for recruitment from a list of all primary day schools within those locations, stratified to ensure representation of both private and public schools, and all eligible geographic zones. The head teachers of the selected schools were invited to an informational meeting and given a brief overview of the study. After securing the head teachers’ agreement to initiate recruitment at their institutions, the head teachers selected 6 schools, 2 from each subcounty, by simple random pick from a bowl.

At each selected school, informational letters were given to a random sample of 24 pupils in grades 5, 6, 7, and 8 in each school, with 6 pupils randomly selected from each grade. These letters described the study and invited parents/caregivers (*parents*) to attend an informational meeting. Additional letters were distributed in 1 of the schools to increase yield.

A total of 7 meetings were held with parents, during which study staff described the nature of the study and answered questions from attendees. In responding to these questions, study staff were able to draw on prepared frequently asked questions (FAQs) documents compiled based on the points raised by parents who had participated in previously conducted FGDs, described below. The recruitment meetings invited parents to volunteer for participation. Study staff individually screened interested attendees for eligibility using a standard form and collected contact information with a view to scheduling enrollment visits. Parents uninterested in enrolling completed a brief questionnaire, identifying reasons for nonparticipation.

### Enrollment

Enrollment took place at the home of potential participants so that they could be easily located in the event of attrition. If a family was not willing to meet at home, an alternative location was chosen. Study staff provided a brief review of the project, answered any remaining questions, and rescreened both parents and preadolescents for eligibility before securing parental consent, followed by child assent. Consent was only required from 1 parent; however, approval of both parents was sought where possible. If more than 1 child in the household was eligible, staff randomly selected 1 for enrollment by drawing a name from a bowl.

**Figure 1:**
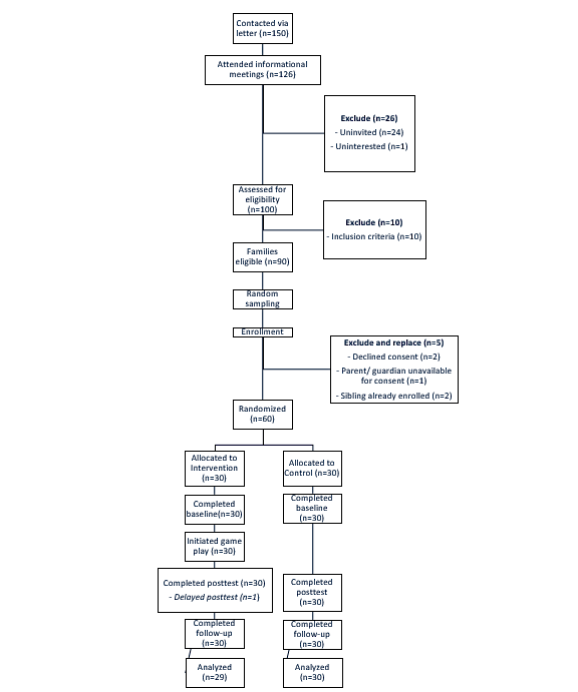
Consolidated Standards of Reporting Trials flow diagram.

In total, 60 parent-child dyads were enrolled, balancing child participants by sex and making efforts to ensure that participation was evenly distributed across the 11- to 14-year age range. This was achieved through stratified sampling of parent/child dyads by age, school, and gender of the child, employing random numbers. Any ineligible or nonconsenting/assenting family was replaced from the pool of other potential participants, taking care to select from the same school where possible and keeping the preadolescents’ age and sex balanced across the whole sample. Parents were informed that if their child was randomly assigned to the intervention group, they would themselves be enrolled and invited to participate in a postintervention focus group. Consents, assents, refusals, and ineligibilities were tracked by study staff at each visit (Figure 1).

### Study Metrics

The primary outcomes of this pilot are measures of study feasibility (recruitment modalities, time to enroll target number of participants, reasons for non-enrollment, participant retention, tracking of study phones, and safety of participants with phones). Participants’ willingness to play the game and the game’s usability, appeal, understandability, relevance, and value are addressed in a separate publication.

The metrics used to assess study feasibility were collected via Excel-based records, assembled by the KEMRI team. These were compiled from paper-based screening forms used during recruitment meetings and consent/assent procedures.

The metrics for game-related primary outcomes were collected via a short multiple-choice assessment of the game experience by the intervention arm participants administered at posttest and by FGDs with them and their parents between posttest and follow-up. In addition, in-game data collection automatically tracked time-stamped user interaction with the interface, allowing for calculations of exposure. Although the data focusing on participants' safety are presented here, findings related to other study metrics will be reported separately.

Data related to secondary (behavioral) outcomes were collected using a survey administered at baseline (T1), immediately following the 16-day intervention (T2), and 6 weeks post intervention (T3). Both this and the game experience survey were implemented via Audio Computer-Assisted Self-Interview software (ACASI, Tufts University) using headphones to ensure participants’ privacy. Outcome data for behavioral mediators are reported elsewhere [27].

### Survey Instrument

The behavioral survey instrument assessed mediators associated with age at onset of sexual activity and condom use at first sex, including knowledge, self-efficacy, risk assessment, perceived social norms, attitudes, behavioral intentions, future orientation, and parent-child dialogue. Thematic domains included puberty, sex, relationships, peer pressure, condom use, HIV, sexually transmitted infections, pregnancy, and alcohol and drugs. These formed the study’s secondary outcomes. We drew individual measures from existing instruments, adapted them for age, linguistic, and cultural appropriateness and consistency of formatting, and we supplemented them with additional measures where necessary. We prioritized existing measures that had been previously used with sub-Saharan African youth populations [38,43-49]. These formed the study’s secondary outcomes. We drew individual measures from existing instruments, prioritizing those previously used with sub-Saharan African youth populations [38, 43-49]. The chosen items were adapted for age, linguistic, and cultural appropriateness [15] and consistency of formatting, and supplemented with additional measures where necessary. In the interest of age appropriateness, we also included hypothetical risk scenarios presented as vignettes [50] that contextualized situational risk assessment, behavioral intention, and self-efficacy. The demographic section of the survey assessing the socioeconomic status was based on Demographic and Health Survey question and response options, reframed to be more meaningful to the young participants [51].

The draft English survey was translated into Dholuo and back translated into English, and subsequently presented to parents to ensure the items would be acceptable to them. Parents participating in these focus groups were also presented with information about the study, and they contributed questions they would want answered if they were considering enrolling their child. Parents’ questions were used as the basis for the FAQ document and informational materials used during recruitment.

Based on feedback received from parents and schools, it was decided that the survey would be delivered solely in English rather than in Dholuo or Kiswahili or with multiple language options. Adding proficiency in Dholuo or Kiswahili as an eligibility criterion would likely have biased our participant sample. Dholuo is not spoken by all Kisumu area residents and Kiswahili, although a national language, was not more consistently understood than English among our participant demographic. As English proficiency was already an eligibility criterion as the intervention was in English, having an English-language survey was not expected to limit the participant pool.

We conducted cognitive assessments of a full draft of the English-language instrument with preadolescents to ensure face validity. Feedback was integrated into the survey between rounds, with each subsequent round. The final survey was programmed into ACASI via the Questionnaire Development System and piloted with 10 preadolescents.

### Staff Training and Informational Materials

The research team had access to a phone loaded with *Tumaini* ahead of the start of recruitment, enabling them to become familiar with the intervention. Recruitment and data collection staff were trained on the game design, study goals, recruitment, enrollment, eligibility screening, and data collection modalities before recruitment and enrollment activities. Staff questions were collected during training and referred to the study leads for clarification. All research team members involved in qualitative data collection had previous experience, including training provided before formative research. This was supplemented with study-specific instructions before all focus groups, to ensure that the specific goals of each round of discussions during survey testing and postintervention FGDs were clear. Additional notes on moderation were provided where needed after review of the discussion transcripts.

Ahead of recruitment and enrollment meetings, informational handouts were developed by the study teams based on parental comments during FGDs and staff questions during training. These were provided to the recruitment and enrollment staff, enabling them to respond to attendee questions and concerns in a uniform way and accurately represent the goals of the study across all meetings. Areas of focus included a review of the consent materials, details of study visits, randomized study arm assignments, and any concerns about the phone, game content, and the logistics of gameplay.

### Randomization

Participants were allocated a study identification number (study ID). A deidentified participant list, including study ID, sex, school, and age, was transmitted to a blinded member of the Emory team, uninvolved with recruitment. From this list, a simple coin toss was used to assign participants to either study arm in a 1:1 ratio, blocking by school and sex.

### Study Procedures

The first study visit, intervention, and second study visit took place during the April 2017 school holiday in Kenya. During the first study visit, at the KEMRI offices, study aims were reviewed, consent and assent procedures were revisited, and the baseline behavioral survey was administered to preadolescents. Participants were given a study card with their assigned study ID for future visits. Upon completion of the survey, the participants were informed to which study arm they had been randomized. Due to the nature of the study, it was not possible for the participants or study team to be blinded. The control arm received standard of care, namely no additional intervention beyond any existing sex education from family, school, and peers.

### Intervention Arm

The intervention arm participants attended a group introductory session after learning their random assignment. Although participants had not known the results of randomization until after the baseline was completed, study staff scheduled each participant’s study visit time slot to ensure that intervention arm participants would all be present on the same day for these sessions. The session covered phone safety, phone and game interface, and instructions about playing the game at least one hour a day for the duration of the 16-day study. Parents of intervention participants were given a short briefing about similar topics, and they were provided with study staff contact information in case a phone- or intervention-related issue arose. These parents were later consented as participants before their participation in postintervention focus groups.

All intervention arm participants were provided with a phone kit containing the study phone, a charger, and a pair of earbuds, to maximize player immersion and ensure that they could have privacy if so desired. Study staff logged phone IDs and phone assignments by participants’ study IDs for future data linkage. The study team had additional phone kits including handsets loaded with *Tumaini* to ensure continued participant activity in the event of a loss, theft, or phone malfunction. Any replacements issued to participants were logged in the participant data management system.

#### Phone Setup

All 30 study phones and 5 additional backup phones were set to local time and current date. All subscriber identification module (SIM) cards were removed and phones fully charged. The game was installed; all other features and apps were blocked using a parental monitoring and control app. This app, enabled and disabled via a passcode, was set to block calling (in the event an active SIM card was inserted), Wi-Fi, and data access, in addition to other apps. These restrictions were set not only to address parents’ concerns that their children might use the phone for other purposes but also to make keeping the phones less attractive to study participants. This was important to ensure that gameplay data could be downloaded from each handset, that ownership of the phone did not act as a disproportionate or coercive incentive, and that all copies of *Tumaini* were collected at the end of the pilot study to ensure equipoise for a future efficacy trial. A simple pattern-based passcode was programmed into all the phones to increase participants’ sense of ownership of the phone and to discourage siblings’ and friends’ unauthorized access. A visual passcode was chosen as being easier to recall than one that was number- or letter-based. All study phones were identified with a unique ID number visible on the outside of the phone and inside the phone case and linked to the participants’ study identifiers in the participant management database. The game was programmed to record the individual phone ID as part of the game’s log file, allowing linkage of gameplay data to individual participants for subsequent analysis.

Before handing out the phones to participants, a daily alarm was set to remind participants to play. The timing was chosen to be as minimally disruptive to the family’s life as possible, with particular consideration to the timing of the study over the Easter weekend.

### Postintervention Procedures

The intervention period lasted 16 days, the maximum length possible during the school holiday period. All 60 participants were contacted by phone to schedule the postintervention visit. Study visit time slots were allocated by study arm as for the baseline visit. All participants took a survey identical to that administered at baseline with the exception that its demographics section was shortened. Intervention arm participants also responded to additional questions about their gameplay experience. The study staff logged the return of phone kits by intervention arm participants and turned off all phones.

The data manager downloaded the gameplay log file from each phone. To allow correct identification and analysis of participants’ data, participants were asked to identify the creator of each profile present in the log file: themselves, a parent (mother or father), a sibling (older or younger, male or female), a friend (male or female), or someone else.

The follow-up survey, identical to the posttest survey, was administered 6 weeks after the posttest.

### Postintervention Focus Groups

Intervention arm preadolescents and their parents participated in FGDs between the postintervention and follow-up surveys. In all, 4 sex- and age-stratified preadolescent groups were held (11-12-year-old females, 11-12-year-old males, 13-14-year-old females, and 13-14-year-old males). Moreover, 4 parent FGDs were held, stratified by the age of their child. All FGDs focused on the appeal of the game, its content, the value of the content and delivery mode, and communication with others about the game. In addition, youth were asked about the appeal of specific components of the game: narrative, characters, mini games, goal-setting component, prizes; their parents were asked for comments on possible future studies. The focus groups took place in a combination of English, Kiswahili, and Dholuo, and were recorded and then transcribed in English.

### Data Analysis

All ACASI data were downloaded from the individual computers by the study staff and saved to a password-protected server in text format. The data manager compiled a master dataset from all 60 entries at each wave of the survey, using Warehouse Manager, a standard data management system used for ACASI. All entries were identified only by participants’ study IDs. All quantitative analyses of data relating to primary and secondary outcomes were carried out using SAS analysis software, version 9.4, using intent-to-treat analysis.

Descriptive statistics of participant characteristics were calculated. Scores from individual survey items with objectively correct or incorrect answers were combined into composite scores by theoretical construct and thematic domain, as well as for the survey overall. Composite scores weighted each question equally on a 0 to 1 scale, with 1 being the *correct* response (eg, high self-efficacy, correct knowledge).

Participants’ change in scores on individual items as well as composite measures between baseline (T1) and posttest (T2), and between T1 and follow-up (T3) were calculated and mean changes (T1 to T2, and T1 to T3) compared across study conditions using 2-tailed 2-sample *t* tests with alpha=.05.

Intervention arm quantitative feedback on the game experience was also imported into SAS and descriptive statistics were calculated and stratified by sex and age group.

Focus group transcripts were loaded into MAXQDA 12 qualitative analysis software (Verbi GmbH). They were coded for themes that emerged inductively from the transcripts (eg, family dynamics) and for deductive themes, based on the discussion guide.

## Results

Recruitment and enrollment were carried out between March 20, 2017 and April 7, 2017. A total of 7 informational meetings were held for parents, with 126 attendees screened for interest and eligibility (Figure 1). Consent and assent procedures were completed in 3 days. One participant who had consented but would not be able to complete all study visits was replaced from the pool of available eligible participants. Participants were age- and sex-balanced. All 60 participants who completed the baseline assessment also completed the other 2 surveys. One intervention arm participant completed the posttest several days late and was excluded from posttest (T2) analyses. All intervention arm participants initiated gameplay. In addition, 27 intervention arm participants and 22 of their parents participated in the focus groups. All study phones were returned; there was no loss or theft of, or damage to, the handsets. A few participants reached out to the study team for troubleshooting help; all concerns were successfully addressed and no phones needed replacing. Phone IDs enabled study staff to accurately link gameplay data to participants’ study IDs.

Participant demographics are presented in Table 1. There were no significant differences across the 2 study arms. Smartphone access and experience were slightly higher in the intervention arm, with only 3 reporting no one in their household having a smartphone and 22 having previously used one. Intervention arm parents participating in the FGDs were mostly female (n=25); only 5 fathers or other male guardians participated.

No safety concerns were reported by parents or participants. All but 1 participant (n=29, 97%) said he or she felt *very safe* while in possession of the phone. The remaining participant indicated he felt “a little safe”—the middle anchor of the Likert scale. During FGDs, parents confirmed that they had not had concerns about their children’s personal safety. In particular, in 3 FGDs, parents attributed their lack of worry to the fact that “it was not a phone that was being used for communication, it was only the game,” (parent of a 11-12-year old).

Preliminary analysis of gameplay log files indicated that across all the profiles participants identified as their own they played, on average, just under 27 hours over the 16 days of the pilot study. Review of log files showed unexpected date and time changes occurring during gameplay. Although the time and date had been set accurately by the study staff, 18 logs (of 30) showed the date and time resetting to their defaults at least once during the study period. These changes affected neither the game’s ability to log player activity nor the ability of data analysts to assess gameplay duration. In addition, parents in 2 FGDs noted that the alarm seemed to ring at inconvenient moments, such as at night or when the child was not nearby to silence it. It seems likely that the changes in time, date, and alarm schedule were linked to phone batteries being removed to attempt to install a SIM card or to charge the battery externally. With the high level of engagement with the game, the alarm reminder is likely unnecessary for a future study.

**Table 1:** Participant demographics at baseline.

Characteristics		Intervention (n=30)	Control (n=30)	Total (N=60)
**Sex, n (%)**				
	Female	14 (47)	16 (53)	30 (50)
	Male	16 (53)	14 (47)	30 (50)
Age (years), mean (SD)		12.8 (1)	12.6 (1)	12.7 (1)
**Religion, n (%)**				
	Catholic	14 (47)	14 (47)	28 (47)
	Protestant/Anglican	8 (27)	2 (6)	10 (17)
	Muslim	2 (7)	4 (13)	6 (10)
	Seventh Day Adventist	4 (13)	4 (13)	8 (13)
	Other	2 (7)	6 (20)	8 (13)
**Living situation, n (%)**				
	Both parents	22 (73)	20 (67)	42 (70)
	One parent	6 (20)	5 (17)	11 (18)
**Housing type, n (%)**				
	Permanent	8 (27)	13 (43)	21 (35)
	Semipermanent	11 (37)	6 (20)	17 (28)
	Temporary	9 (30)	6 (20)	15 (25)
	Iron sheets	2 (7)	4 (13)	6 (10)
**Smartphone ownership, check all that apply, n (%)**				
	Parent	21 (70)	15 (50)	36 (60)
	Self	2 (7)	1 (3)	3 (5)
	Sibling	11 (37)	5 (17)	16 (27)
	Other adult	4 (13)	1 (3)	5 (8)
	No one	3 (10)	8 (27)	11 (18)
Have used a smartphone before baseline, n (%)		22 (73)	19 (63)	41 (68)

Additional results related to acceptability and to the study’s secondary (behavioral) outcomes were analyzed, and these are reported in separate [27] and forthcoming publications.

## Discussion

### Principal Findings

This manuscript describes the pilot study of a smartphone-based HIV prevention intervention for preadolescents in Kenya. Theoretically grounded mobile interventions, particularly those delivered via smartphone, have the potential to engage young people and effect behavior change without requiring a large cadre of project staff to deliver, maintain, and update the content. In particular, well-designed smartphone-based interactive games can provide opportunities to learn, practice, and strengthen crucial health-protective skills in a safe space.

This pilot study shows the feasibility of a trial of a smartphone intervention in which the game is delivered on study-provided phones that were subsequently collected. Although smartphone ownership is increasing and handsets have become more affordable, ownership does still skew toward the wealthier [52]. If smartphone ownership or access had been an eligibility criterion, the sample would have been biased toward a higher socioeconomic status, an important factor in the team’s decision to provide smartphones to study participants. Projected increases in smartphone ownership over time (by 2025, mobile phone penetration is expected to reach 52% in sub-Saharan Africa as a whole, up from 44% in 2017, with 67% of connections expected to be via smartphone) [17] will support roll out of the game-based intervention, should it prove efficacious. At 80% of the adult population, Kenya mobile phone ownership is higher than that of sub-Saharan Africa as a whole [53]. It is also expected that if the game is rolled out, it is likely to be accessed on a parent’s or older sibling’s phone rather than on an adolescent’s own device. In the meantime, this model of intervention delivery offers an important and feasible means to reduce sociodemographic bias and ensure consistency of technology when testing smartphone-based mHealth interventions.

This pilot study explored recruitment initiated through schools, made possible by securing approval from the local Ministry of Education before contacting school officials. By engaging the Ministry of Education early in the study process, the team was able to address any potential concerns and secure buy-in, crucial to carrying out research activities with a youth population. In addition, the study team engaged parents throughout the study, including providing informational sessions to assess their interests in participating and to answer questions before study activities. We believe this helped to establish transparency with parents and increase their willingness to participate and encourage their children to remain in the study, contributing to rapid enrollment and a lack of attrition. These recruitment and engagement strategies could be scaled up successfully for a future larger-scale randomized trial of this and similar interventions. Zero loss to follow-up and a lack of safety concerns support the feasibility of this type of study and its potential for scale-up.

### Future Directions

A future efficacy trial is likely to take the form of a multiyear RCT, with repeated behavioral measures supplemented with collection of a herpes simplex virus 2 (HSV-2) biomarker. HSV-2 is relatively prevalent and, as such, has been used in conjunction with self-reported sexual behavior in other studies of interventions aimed at reducing HIV infection among young people in sub-Saharan Africa [54]. Additional protocols for disclosing test results and linking to care will need to be developed in line with Kenyan guidelines for HIV testing [55].

The larger sample necessitated by the proposed efficacy trial will require an expanded recruitment strategy from a larger pool of primary schools. There are over 200 primary education establishments in the Central, East, and West Kisumu subcounties; therefore, this is not expected to limit our ability to recruit the necessary pool of participants, even without expanding the recruitment area.

In view of the increased sample size, we will consider shifting survey delivery to a mobile platform, such as Open Data Kit on phones or tablets. This would allow faster survey implementation via simultaneous delivery at multiple sites across Kisumu without losing the audio component of ACASI (important to maximize comprehension).

### Limitations

Due to the length of the school holiday period during which the trial took place, the intervention period was limited to 16 days and follow-up activities took place over 6 weeks. During a full-scale efficacy trial, the expected exposure to *Tumaini* will be more extensive and will allow for a more thorough assessment of player engagement and game use over time. This assessment will also allow us to determine any potential associations between length of exposure and outcomes and thereby estimate a minimum recommended exposure time. The game was designed with a minimum exposure time of 10 to 12 hours in mind; this corresponds to the approximate duration of a group-based intervention. If study results indicate that wider distribution of the intervention is warranted, no external limit to play time would be placed on users.

Although this study did not experience any attrition, we acknowledge that this may be related to the short duration of the intervention period and its follow-up assessments and that a longer, multiyear efficacy study is likely to lead to some loss to follow-up (KEMRI studies indicate that typical attrition is 15% over the course of a multiyear study). The KEMRI team has a strong track record of retaining participants during longitudinal studies, including through intermittent contact with participants between study visits and repeated contact ahead of visit scheduling.

### Conclusions

The findings of this pilot indicate that it is feasible to safely test a game-based HIV prevention intervention delivered via smartphone, with effective recruitment and no issues of loss to follow-up or of study-provided smartphones. These methods will inform the development of a larger RCT of the *Tumaini* game-based intervention. This study provided intervention arm participants with low-cost smartphones on which the game was preloaded. If this intervention were to prove efficacious, it is anticipated that increases in smartphone ownership would support rollout at scale. The approach tested here has the potential to guide the development of other smartphone-based mHealth intervention studies aimed at school-aged populations in low-resource settings.

## Appendix

Multimedia Appendix 1 Sample Tumaini graphics.

## Abbreviations

ACASI: Audio Computer-Assisted Self-Interview
FAQ: frequently asked question
FGD: focus group discussion
HSV-2: herpes simplex virus 2
ID: identification
KEMRI: Kenya Medical Research Institute
mHealth: mobile health
RCT: randomized controlled trial
SIM: subscriber identification module
