# Supplementary material for: A Smartphone Game to Prevent HIV Among Young Africans: Protocol for a Randomized Pilot Study of a Mobile Intervention
Source: JMIR Res Protoc. 2019 Mar 27;8(3):e11209. doi: 10.2196/11209 (PMC6456823; doi:10.2196/11209)

<u>Multimedia Appendix 1.</u>

<u>Sample *Tumaini* graphics</u>

<u>Sample graphic from narrative</u>

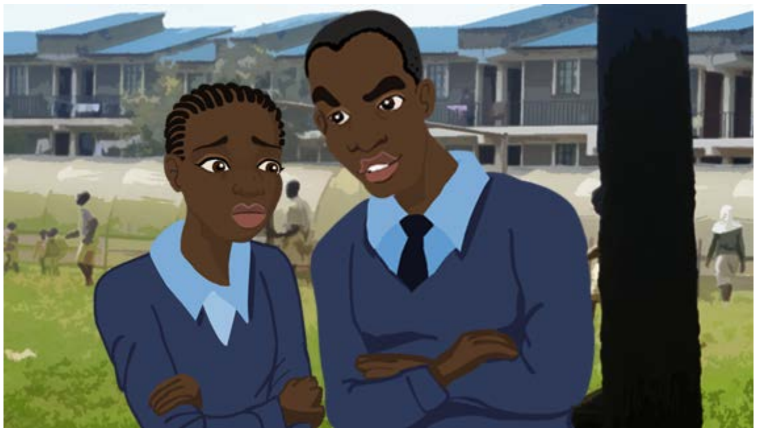

<u>Sample graphic from mini-games</u>

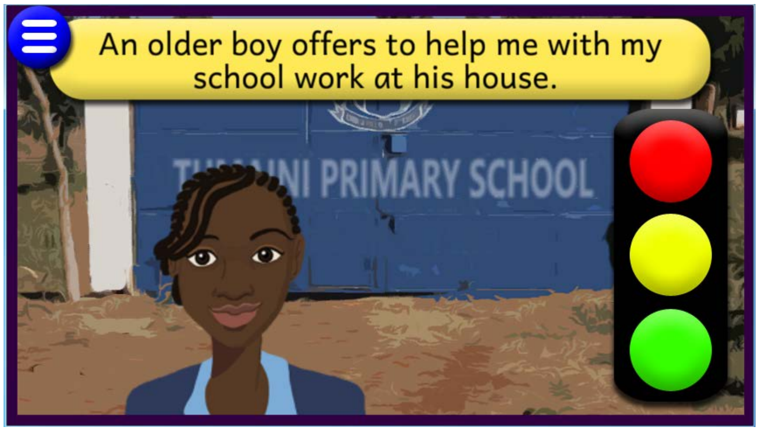

<u>Sample graphic from My Story</u>

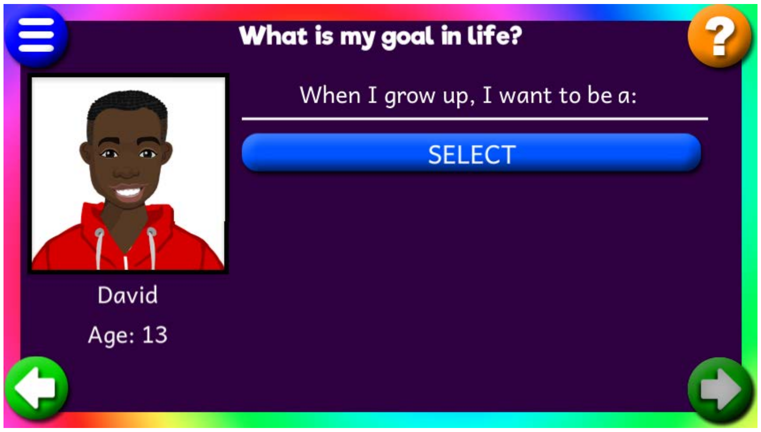

Supplement: Multimedia Appendix 1 [file resprot_v8i3e11209_app1.pdf]
